# Acute thrombotic occlusion of a profunda femoris artery aneurysm

**DOI:** 10.1093/jscr/rjag026

**Published:** 2026-01-30

**Authors:** Tomoki Nishimura, Hiromitsu Nota, Keiji Matsubayshi

**Affiliations:** Department of Cardiovascular Surgery, Omihatiman Community Medical Center, Tsuchidacho, Omihachiman, Shiga 523-0082, Japan; Department of Cardiovascular Surgery, Omihatiman Community Medical Center, Tsuchidacho, Omihachiman, Shiga 523-0082, Japan; Department of Cardiovascular Surgery, Omihatiman Community Medical Center, Tsuchidacho, Omihachiman, Shiga 523-0082, Japan

**Keywords:** profunda femoris artery aneurysm, common femoral artery aneurysm, thrombosis, superficial femoral artery, surgical intervention

## Abstract

A profunda femoris artery aneurysm (PFAA) is an extremely rare peripheral arterial lesion, often first detected following rupture or swelling of the groin or thigh region. We report a 74-year-old man who presented with acute thigh pain caused by thrombotic occlusion of a left PFAA associated with a common femoral artery aneurysm (CFAA). Contrast-enhanced computed tomography at presentation demonstrated complete left PFAA thrombosis and partial thrombus formation within the left superficial femoral artery. The aneurysms’ diameters were 30 mm for the left CFAA and 35 mm for the left PFAA. The patient’s symptoms stabilized after systemic heparinization. Considering the recurrent thrombosis and potential aneurysmal rupture risks, an elective surgical intervention was performed. This procedure consisted of PFAA ligation, prosthetic graft replacement of the CFAA, and lateral circumflex femoral artery branch reconstruction. The patient’s postoperative course was uneventful, and follow-up imaging confirmed excellent graft patency and preserved thigh perfusion.

## Introduction

Profunda femoris artery (PFA) aneurysm (PFAA) is a rare vascular disorder, representing ~ 0.5% and 1%–2.6% of peripheral and femoral aneurysms, respectively [1, 2]. Because the PFA lies deep within the thigh musculature, it is often asymptomatic and difficult to detect early. According to previous reports, PFAAs were incidentally detected in 34.8% of cases, whereas thrombosis and rupture occurred in 6.9% and 27.9%, respectively. In addition, 58% of patients had other concomitant arterial aneurysms [2, 3]. Thromboembolic occlusion of PFAAs is extremely rare, and in a systematic review by Kibrik *et al.* (2020), such cases were infrequently reported [1]. We present a rare case of thromboembolism in a PFAA that was successfully treated with aneurysm resection, prosthetic graft replacement, and lateral circumflex femoral artery branch reconstruction to preserve thigh perfusion.

## Case report

**Figure 1:**
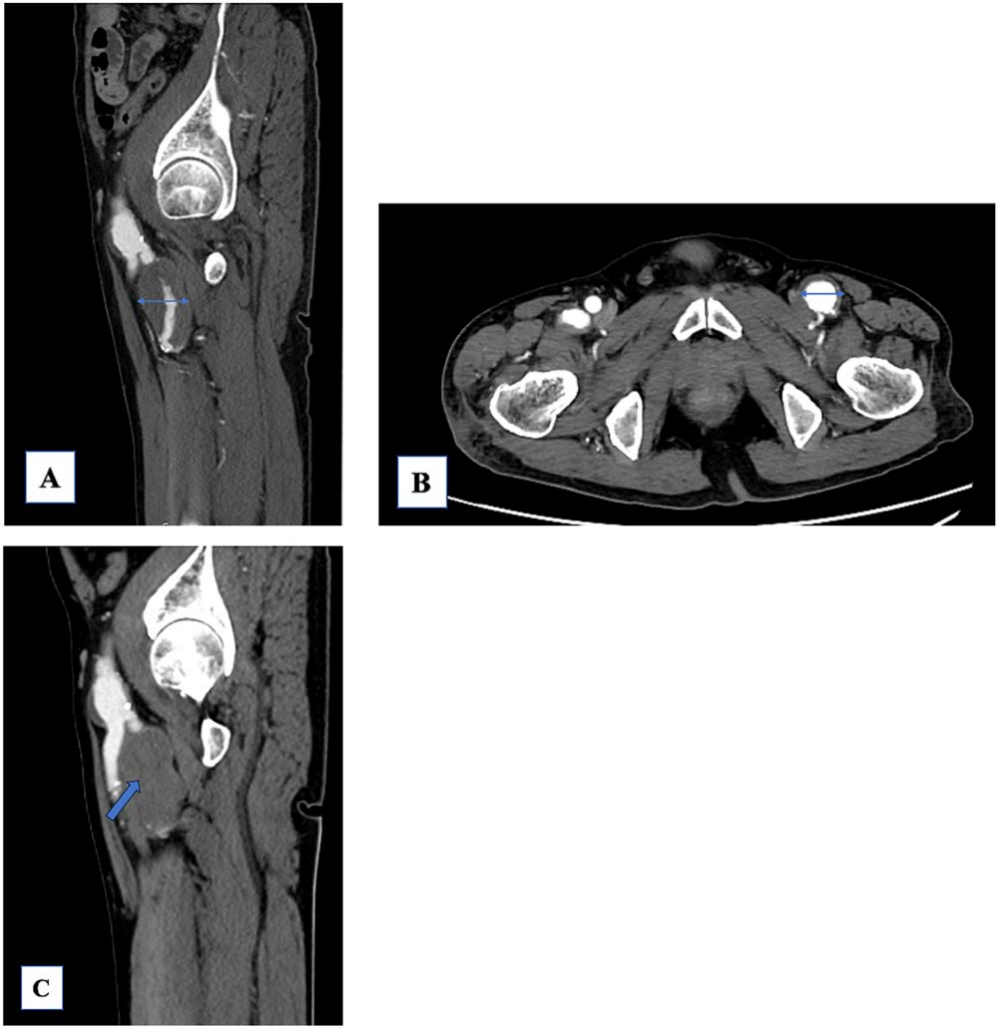
**(**A) The left profunda femoris artery aneurysm (PFAA) measures 32 mm. A large thrombus is observed. The peripheral vessels are patent. (B) The common femoral artery aneurysm (28 mm) shows a mural thrombus, and the left PFAA is enlarged to 35 mm. (C) The PFAA is completely occluded by the thrombus.

**Figure 2:**
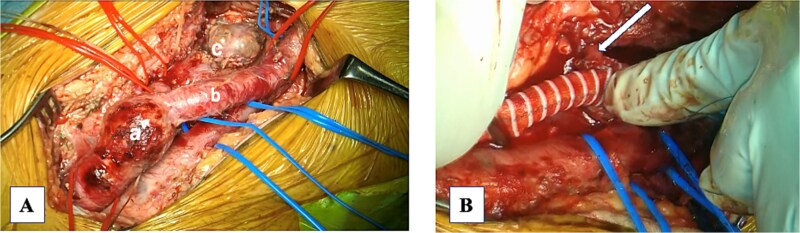
Intraoperative photograph. (A) Common femoral artery aneurysm (CFAA): (B) superficial femoral artery. (C) Profunda femoris artery aneurysm (PFAA). (B) The CFAA was replaced with a prosthetic vessel, and the PFAA was resected proximally. A branch of the lateral circumflex artery was anastomosed to the prosthetic vessel (arrow).

**Figure 3:**
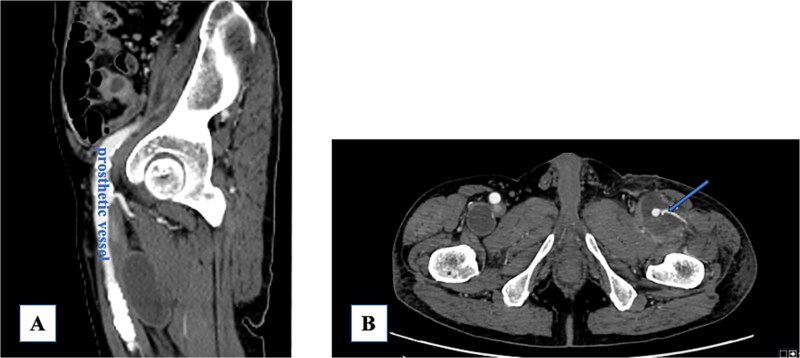
(A) Post-prosthetic vessel replacement. (B) The lateral circumflex artery is patent (arrow).

A 74-year-old man with known bilateral PFAAs (left: 32 mm; right: 38 mm) and a left common femoral artery (CFA) aneurysm (CFAA) (28 mm) (Fig. 1A and B) was referred to our department because of sudden-onset left thigh pain and inability to walk. Physical examination revealed left thigh swelling and livedo reticularis. Left popliteal and pedal pulses were palpable. Contrast-enhanced computed tomography (CT) demonstrated acute thromboembolism within the left PFAA, with complete distal PFA occlusion; the left PFAA had enlarged to 35 mm, and a thrombus was present within the left superficial femoral artery (SFA); however, the distal vessels showed contrast enhancement (Fig. 1C). ECG showed sinus rhythm. Echocardiography showed no intracardiac thrombus and preserved cardiac function. The ankle-brachial index was normal. Laboratory data revealed elevated creatine kinase levels (peak = 21 675 U/L) and a mild increase in the erythrocyte sedimentation rate, with otherwise normal renal function. After 5 days of systemic heparinization, elective surgery was performed under general anesthesia. We performed surgery to prevent recurrent SFA thrombosis and to avoid potential PFAA rupture. Surgery was deferred for 5 days because the pre-existing collateral circulation appeared well developed, allowing symptomatic improvement with systemic heparinization and preventing progression to thigh muscle necrosis. During this interval, the creatine kinase levels peaked and then dropped, accompanied by thigh pain relief and recovery of ambulation. Subsequently, the patient underwent aneurysm resection under stable hemodynamic and metabolic conditions. A 15-cm longitudinal incision was made in the left inguinal region. The CFA, SFA, and PFA were exposed and several branches were ligated (Fig. 2A). A lateral circumflex femoral artery branch arising near the CFA bifurcation showed strong backflow; therefore, reconstruction of this branch was planned to preserve lateral thigh perfusion. The PFAA was opened, revealing a large organized thrombus with no backflow from the distal vessels. As reconstruction of the peripheral PFA was technically difficult, we performed complete PFAA resection and replaced the CFAA with an 8-mm straight prosthetic graft (Intergard K; Cosmotec, Tokyo, Japan). A lateral circumflex femoral artery branch was anastomosed to the prosthetic graft (Fig. 2B). The postoperative course was uneventful, and the patient regained independent ambulation. Contrast-enhanced CT confirmed satisfactory perfusion (Fig. 3A and B). The patient was discharged on postoperative Day 14.

## Discussion

PFAA is an uncommon peripheral arterial lesion, accounting for <1% of femoral aneurysms, and is typically caused by atherosclerotic degeneration or as a pseudoaneurysm resulting from iatrogenic injury or trauma [2, 3]. Due to its deep location, it often presents only after symptoms such as rupture, venous compression-induced edema, groin swelling, or thrombosis [2–4]. In a systematic review by Kibrik et al., only a few PFAA cases complicated by thrombotic occlusion were identified among the published reports, underscoring its rarity and lack of established management strategies [1]. Regarding size, elective repair is recommended for PFAAs measuring ≥35 mm, and this criterion applied to our patient [5]. The PFA is crucial in the lower limb’s collateral circulation, especially when the SFA is stenotic or occluded [4]. Various surgical options have been reported, including ligation, aneurysmectomy, and reconstruction; however, open repair with revascularization is generally recommended because of the complex branching anatomy and the need to preserve deep thigh collateral flow [1, 2, 4, 6]. In this case, the aneurysm was completely thrombosed and lacked distal backflow, making direct anastomosis of the distal PFA technically impossible. Instead, we reconstructed the lateral circumflex femoral artery branch, providing strong retrograde flow and maintaining thigh perfusion.

Although thrombotic PFA occlusion interrupts distal flow, Shintani et al. reported no ischemic complications after simple proximal ligation in three cases [7]. This suggests that collateral circulation may sufficiently maintain perfusion in some patients. In our case, the thrombus was limited to the PFA, and the symptoms improved rapidly with heparin alone, implying that urgent surgery was not initially required. Given that the SFA remained patent, the collateral channels to the distal PFA territory may have prevented critical ischemia. However, as previous studies have demonstrated, simple ligation or an untreated aneurysm may lead to late complications such as limb loss [3, 8, 9]. In addition, simple ligation without revascularization may expose the aneurysm to retrograde blood flow, potentially resulting in further enlargement. Therefore, although the patient’s symptoms stabilized with systemic heparinization, surgical intervention was still warranted. Several reports have described endovascular PFAA repair, including stent graft placement and endovascular embolization. These endovascular approaches offer significant advantages such as minimal invasiveness and easy target vessel access [10, 11]. However, the PFA gives rise to multiple muscular branches that provide crucial collateral flow to the lower extremities, and inadvertent occlusion of these branches during endovascular exclusion may result in thigh ischemia [3, 11, 12]. Our case highlights thrombotic PFAA occlusion’s rarity, for which published management strategies remain limited. When thrombosis alone is present, systemic heparinization may be sufficient to stabilize the acute phase, and urgent surgical revascularization is not always required. However, once the patient’s condition stabilizes, elective aneurysm resection with appropriate distal revascularization represents a reasonable and definitive treatment strategy. In conclusion, we report an extremely rare acute PFAA thrombosis case. When thrombosis is limited to the PFA alone, urgent surgery may not be necessary because symptoms can stabilize with anticoagulation. Elective aneurysm resection with appropriate revascularization offers favorable long-term outcomes.

## Data Availability

No new data were generated in this research.
